# The mitochondrial intermembrane space: the most constricted mitochondrial sub-compartment with the largest variety of protein import pathways

**DOI:** 10.1098/rsob.210002

**Published:** 2021-03-10

**Authors:** Ruairidh Edwards, Ross Eaglesfield, Kostas Tokatlidis

**Affiliations:** Institute of Molecular Cell and Systems Biology, College of Medical, Veterinary and Life Sciences, University of Glasgow, University Avenue, Glasgow G12 8QQ, UK

**Keywords:** mitochondria, protein import, oxidative protein folding, intermembrane space

## Abstract

The mitochondrial intermembrane space (IMS) is the most constricted sub-mitochondrial compartment, housing only about 5% of the mitochondrial proteome, and yet is endowed with the largest variability of protein import mechanisms. In this review, we summarize our current knowledge of the major IMS import pathway based on the oxidative protein folding pathway and discuss the stunning variability of other IMS protein import pathways. As IMS-localized proteins only have to cross the outer mitochondrial membrane, they do not require energy sources like ATP hydrolysis in the mitochondrial matrix or the inner membrane electrochemical potential which are critical for import into the matrix or insertion into the inner membrane. We also explore several atypical IMS import pathways that are still not very well understood and are guided by poorly defined or completely unknown targeting peptides. Importantly, many of the IMS proteins are linked to several human diseases, and it is therefore crucial to understand how they reach their normal site of function in the IMS. In the final part of this review, we discuss current understanding of how such IMS protein underpin a large spectrum of human disorders.

## Graphical abstract

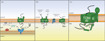

## 1. Introduction

The mitochondrion is a relatively small yet complex organelle responsible for a plethora of cellular activities, the production of 95% of the cell's ATP being just one of them. Mitochondria are key players in apoptosis, phospholipid biosynthesis, haem biosynthesis and calcium homeostasis [1]. ‘The powerhouse of the cell’ has an outer and an inner membrane separating two aqueous sub-compartments, the matrix and intermembrane space (IMS). The IMS is the smallest of the two aqueous sub-compartments (the other one being the innermost matrix of mitochondria), but it is responsible for several of the aforementioned functions within mitochondria. 99% of the proteins that reside in mitochondria are nuclear-encoded and have to be imported into the organelle via different protein translocases and import complexes that direct each protein to the correct compartment. All of the 51 yeast and 53 human IMS proteins are nuclear-encoded [2–4] and hence imported from the cytosol. The difference in the annotation of 127 proteins as human IMS proteins in MitoCarta 2.0 [2] arises from (i) the fact that IMS-APEX2 proximity biotinylation used in [3] labels IMS as well as these in the mitochondrial OM and IM that are accessible to the IMS biotin label, and (ii) the fact that some proteins that are in the IMS during their biogenesis have been annotated in [4] as IM proteins if they mature to be part of a complex in the IM. Interestingly, the IMS has numerous distinct import pathways, in contrast to the mitochondrial matrix that is reached by mitochondrial preproteins following a single default import pathway.

In this review, we discuss each of the several IMS import pathways including the Mia40, cytochrome *b*2, cytochrome *c*, cytochrome *c* haem lyase (CCHL) and UCP import pathways. Furthermore, we highlight some of the atypical IMS import pathways and speculate on the as yet unknown IMS import pathways. The IMS contains many rather small but structurally distinct proteins that are implicated in several human diseases including several mitochondriopathies, amyotrophic lateral sclerosis (ALS), Parkinson's disease and Alzheimer's disease (AS). The roles of dysfunctional mitochondrial IMS proteins in underpinning these diseases are also discussed in this review.

## 2. The Mia40 import pathway

A large portion of the 51 IMS proteins in yeast (53 in humans) are small in size and contain no N-terminal mitochondrial targeting sequence [2,3]. Instead, many of them possess twin cysteine motifs typically of CX3C and CX9C configurations which mould the structural characteristics of these proteins that fold in a helix-turn-helix structure with two structural cysteine disulfide bonds connecting the CX3C or CX9C motifs between the two helices [5–7]. These proteins are imported and folded in the IMS via an oxidative folding system (also called a disulfide relay system), the key components of which are the oxidoreductase Mia40 and sulfhydryl oxidase Erv1 [7–12].

**Figure 1.**
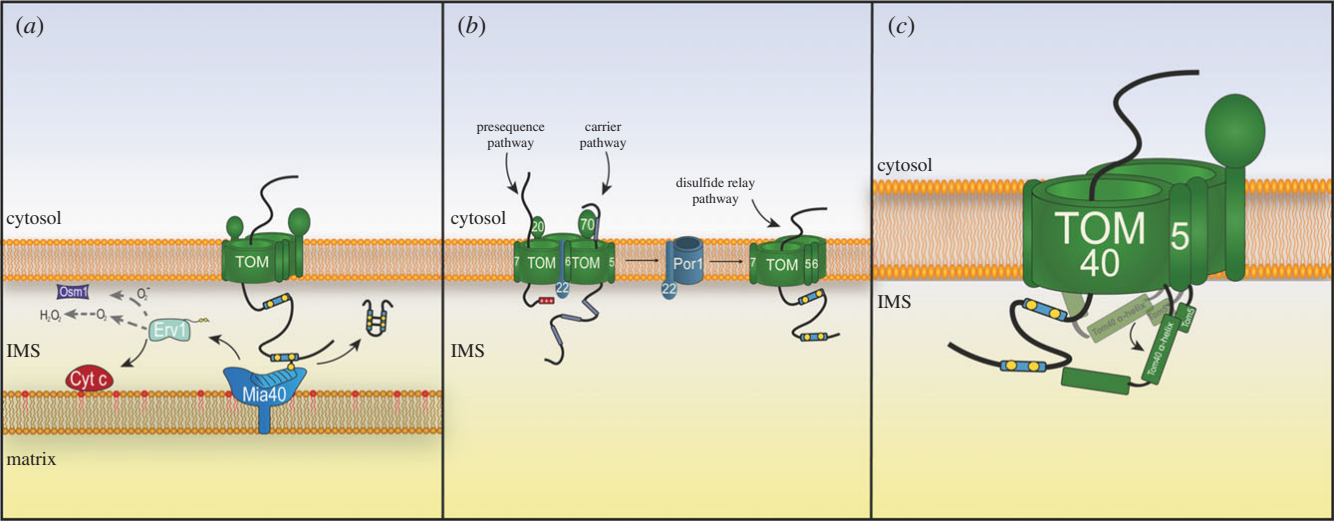
The import of Mia40 substrates. (*a*) Import of Mia40 substrates proteins first requires translocation through the outer membrane TOM complex. The cysteine containing intermembrane space targeting signal (ITS) subsequently binds to Mia40 via a disulfide intermediate. This intermolecular disulfide bond is substituted for an intramolecular disulfide bond within the substrate protein. Mia40 is then re-oxidized via an electron transfer reaction from the sulfhydryl oxidase Erv1. Erv1 can transfer electrons to cytochrome *c*, H_2_O_2_ or Osm1 under anaerobic conditions. (*b*) The TOM complex can be found in two oligomeric states, trimeric and dimeric. The trimeric complex is the predominant complex and preferentially imports of matrix and inner membrane proteins. The dimeric complex is thought to favour Mia40 substrate protein import. This dimer–trimer TOM complexes are regulated by the small Tom protein Tom6 and the voltage gated anion channel, Porin. Tom6 retains the core Tom receptor Tom22 within the trimeric TOM complex while Porin is responsible for binding Tom22 to facilitate the formation of the dimeric TOM complex. (*c*) Tom5 is responsible for manoeuvring the N-terminal α-helix of Tom40 which occludes the major pore of the translocase out of the way to facilitate the import of Mia40 substrates into the IMS.

In *Saccharomyces cerevisiae*, Mia40 is an inner membrane anchored protein that faces the aqueous IMS. Interestingly, in metazoans Mia40 (CHCHD4 in humans) is a soluble protein found in the bulk IMS. CHCHD4 still remains near the inner membrane like in yeast, as it interacts with the protein apoptosis inducing factor 1 (AIFM1). AIFM1 is also responsible for the import of CHCHD4 via the N-terminus of CHCHD4, which interacts with the dimeric form of AIFM1 [13,14]. The substrate binding domain of Mia40 is highly conserved from yeast to humans and comprises 6 cysteine residues, four of which contribute to the folded helix-turn-helix structure of the protein, while the remaining two are responsible for its function [7,15]. This redox-active semi-oxidized ‘CPC’ motif binds covalently the substrate proteins after their translocation through the translocase of the outer membrane (TOM) complex in an unfolded and reduced state [16]. An intermolecular disulfide bond is formed between the substrate protein and the second cysteine of the CPC motif of Mia40 [7,17]. This second cysteine of Mia40 is then substituted via nucleophilic attack for the second cysteine residue in the substrate protein thus creating an intramolecular disulfide bond within the substrate imported protein [7,16,18,19]. This is followed by release of the folded substrate protein into the IMS (figure 1*a*). Interestingly, both Mia40 and Erv1 require Mia40 for import and folding [20,21]. Erv1 is imported and folded by Mia40 via its CX16C motif which is critical for its structure [21]. Erv1 has three cysteine motifs, closest to the N-terminus is a shuttle cysteine motif which is responsible for interaction and reoxidation of Mia40. A second catalytic cysteine motif binds the cofactor flavin adenine dinucleotide (FAD) and the structural CX16C is the final cysteine motif. The N-terminal shuttle motif is not required for its import. On the other hand, yeast Mia40 is imported in an unconventional manner. Mia40 possesses an N-terminal signal stop-transfer sequence that is imported via the Tim23 complex whereby Tim23 integrates Mia40 into the inner membrane. Endogenous Mia40 is then required for the oxidation and folding of the functional IMS domain of Mia40 [20].

In order for Mia40 to continually function, recycling is required via an electron transfer reaction. Initially the shuttle cysteine motif of Erv1 re-oxidizes Mia40 [10,11,21]. The electrons are then shuttled from this motif to a central CXXC motif within Erv1 and then onto FAD which is non-covalently bound to Erv1 [21–23]. Completion of the reaction requires transfer of the electrons to a final acceptor. To fulfil this role, there are four known terminal electron acceptors. The typical electron acceptors are cytochrome *c*, cytochrome *c* oxidase and cytochrome *c* peroxidase [24–27]. Erv1 can also transfer its electron directly to molecular oxygen which subsequently generates hydrogen peroxide (H_2_O_2_). Under anaerobic conditions Erv1 is known to transfer the electrons to Osm1 and fumarate [28]. Erv1 has been shown to be an efficient enzyme under normal conditions but it contributes to H_2_O_2_ production within the IMS and it also has a tendency aggregate, particularly at elevated temperatures [10,29]. This Erv1 dysfunction results in degradation by the IMS protease Yme1 and could potentially distort the redox balance of Mia40 resulting in decreased substrate protein import [29]. The yeast thiol peroxidase Gpx3 was recently found to be capable of re-oxidizing Mia40, suggesting other means of maintaining the redox state of Mia40 in a variety of conditions [30]. Further studies are required to determine if other proteins are capable of re-oxidizing Mia40 and whether other electron acceptors exist. There are two accounts of proteins responsible for maintaining optimal activity of Mia40. The zinc binding protein Hot13 is capable of chelating zinc ions to enable efficient reoxidation of Mia40 [31]. In addition to this, the glutaredoxin system is involved in the maintenance of the redox state of Mia40 and is thought to be involved in Mia40 substrate proofreading as it prevents stalling of substrate proteins covalently attached to Mia40 via reduction and retro-translocation [32–34]. Mia40 substrate retro-translocation has been reported whereby the intermolecular disulfide bond is reduced and the substrate proteins are retro-translocated through the TOM complex back out into the cytosol where they are degraded by the proteasome [34,35]. In yeast cells, the critical reduction step was effected by the addition of chemical reductants [34]. However, in human cells overexpression of the protein glutaredoxin1, a reducing protein found in the IMS, showed that this retro-translocation probably occurs *in vivo.* The two critical regulators of such an event are the length of time the substrate is covalently attached to Mia40 and the redox state of the IMS [33]. The balanced effect of these two determinants results in either successful disulfide bond formation and retention of the substrate in the IMS or retro-translocation and degradation in the cytosol. In yeast, the glutaredoxin or thioredoxin system could potentially fulfil this reducing role and facilitate retro-translocation of Mia40 substrates in the event of substrate protein stalling.

## 3. Mia40 substrate translocation and recognition

Mia40 substrates are translated on cytosolic ribosomes and imported via the TOM complex. Until recently, the mechanistic aspects of this import process were not fully understood. Mia40 substrates do not require the TOM complex receptors Tom20, Tom22 and Tom70 which 95% of mitochondrial proteins depend on for their import [2,36,37]. Instead it has been shown that the TOM receptor Tom5 is involved in the import of Mia40 substrates [37,38]. The recent elucidation of the atomic structure of the TOM complex by cryo-EM shed light on the reasons why this is the case [38,39]. The channel-forming core subunit of the TOM complex, Tom40, has an N-terminal α-helical extension that faces the IMS and partially blocks the trans side of the Tom40 pore [38,39]. This extension interacts with Mia40 and Tom5 to facilitate early stage substrate translocation and docking with Mia40 (figure 1*c*) [38]. Secondly, the positioning of the N-terminal α-helical extension is critical for the late stage complete import and folding of the substrate protein within the IMS [38]. This aspect is controlled by key residues within Tom40 itself. Combined, this highlights a divergent translocation mechanism for Mia40 substrates compared to matrix and inner membrane targeted proteins that are imported through the TOM complex in a different manner. Furthermore, substrates of Mia40 are thought to engage for import via a small population of *dimeric* TOM complexes [40–43]. This differs from all the other import pathways which engage via the more abundant *trimeric* TOM complex. The dynamic *dimer–trimer* transition of the TOM complex is regulated by the Tom6 receptor which stabilizes the Tom22 receptor within the trimeric TOM complex while the metabolite transporter porin interacts with Tom22 to allow the formation of a dimeric TOM complex [43]. The *trimeric* complex that contains Tom22 is more suited to the import of matrix presequence and inner membrane carrier proteins. The *dimeric* TOM complex has only been shown to be important for the import of Mia40 substrates so far (figure 1*b*). A possible reason why Mia40 substrates favour the dimeric TOM complex over the trimer is because of the bulky IMS domain of Tom22 that interacts with the translocase of the inner membrane subunit, Tim50, this transmembrane tether could sterically hinder the import of Mia40 substrates [43,44]. The dimeric TOM complex repositions Tom22 in such a way that it favours the Mia40 interaction with the N-terminal α-helical extension of Tom40 and Tom5 [38]. Further research is required to understand what cytosolic components are involved in the import of Mia40 substrates, something that has already been uncovered for outer and inner membrane proteins [45].

**Figure 2.**
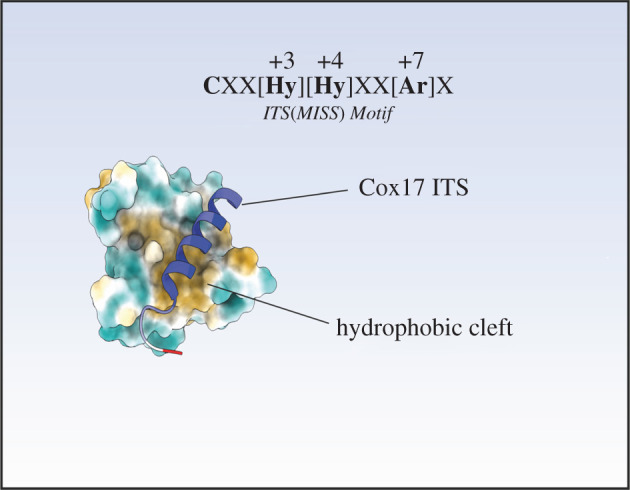
The intermembrane space targeting signal (ITS). The ITS comprises a conserved motif that binds to the hydrophobic binding cleft of Mia40 in a particular way. The hydrophobic residues of the ITS motif interact with specific hydrophobic residues found within Mia40 to orientate the substrate protein in such a way to optimize disulfide bond formation. The structure is from RCSB: 2L0Y [47].

The recognition of Mia40 substrates by Mia40 is governed by internal targeting sequences called IMS targeting signals (ITS) or mitochondrial intermembrane space sorting signals (MISS) [16,46]. These sequences have a propensity to form an amphipathic α-helix with a hydrophobic face that is recognized by the hydrophobic binding cleft of Mia40 (figure 2) [5,18]. The ITS is 9 amino acids in length and can be located upstream or downstream of the docking cysteine. The consensus sequence of ITS is CXX [Hydrophobic] [Hydrophobic]XX [Aromatic]X motif [16]. A combination of mutagenesis, pull-down experiment, and biophysical and structural analysis has shown that the binding of substrate proteins to Mia40 follows a sliding-docking model [13] whereby the precursor is first aligned to the cleft of Mia40 by hydrophobic packing of the ITS in a non-covalent binding reaction (‘sliding’ step). This results in juxtaposition of the docking cysteine of the substrate to the second cysteine of the CPC motif of Mia40, allowing thus these two cysteines to link to an intermolecular disulfide bond (‘docking’ step). It has been shown that the oxidoreductase function of Mia40 (involving the electron transfer reaction) is not essential for the import of Mia40 substrates [17]. Instead the hydrophobic binding cleft of Mia40 acts as a holdase (according to the first non-covalent binding step of the sliding-docking model) that is capable of recognizing ITS signals lacking the docking cysteine residue [17]. This grip on the substrate allows for import (i.e. translocation across the outer membrane), but not folding suggesting that Mia40 acts as a trans-site receptor when importing Mia40 substrates. However, the catalytic CPC motif is essential for creation of the disulfide bond in the substrate, which is a prerequisite for its folding.

## 4. Unconventional Mia40 substrates

Although the vast majority of Mia40 substrates are imported via well conserved specific CX3C and CX9C motifs, there is a growing number of Mia40 substrates that do not have specific such motifs and have been shown to be imported in an unconventional manner. The inner membrane translocase Tim22 contains an unconventional CX98C cysteine motif that is directly oxidized by Mia40 which subsequently results in integration of Tim22 into the IM [48]. The matrix localized mitochondrial ribosomal protein Mrp10 has also been shown to be oxidized by Mia40 on its translocation into the matrix [49]. The N-terminus of Mrp10 has a uncharacteristic proline-rich matrix targeting signal that is thought to aid in the access of Mia40 to the conserved cysteine residues of Mrp10 by avoiding the tethered TOM-TIM23 super complex that conventional matrix proteins use for their import. How the proline-rich region achieves this has yet to be elucidated. The copper chaperone Ccs1 is another atypical Mia40 substrate that forms only a single disulfide bond involving a non-conventional CX36C cysteine motif [50–52]. The import and retention of Ccs1 in the IMS via the MIA pathway in turn affects the levels of the critical superoxide scavenger Cu–Zn superoxide dismutase Sod1 within the IMS [50–52]. The metalloprotease Atp23 is one of the largest known Mia40 substrates at 27 kDa and has 10 cysteine residues. All five cysteine pairs appear to be oxidized by Mia40 raising the potential for isomerase activity of Mia40, since the introduction of non-native disulfide bonds is likely in a protein with five disulfide bonds [53]. Future studies will hopefully shed light on whether Mia40 has, at least for some substrate proteins, such protein disulfide isomerase activity. Finally, post-translational modification plays a role in the import of the Mia40 substrate Mic19, a component of the mitochondrial contact site and cristae organizing system (MICOS) responsible for the maintenance of inner membrane-outer membrane contacts and crista junctions [54]. This protein contains a single CX10C motif and a large domain of unknown function (DUF). The DUF domain is thought to affect accessibility of the CX10C motif during import. This hinderance is circumvented by the post-translational addition of a myristoyl group at the N-terminus of the polypeptide. This myristoyl group is recognized by the Tom20 import receptor and is thought to aid in the import of the bulky DUF domain of Mic19 allowing suitable exposure of the CX10C motif to Mia40 [54]. Further studies will hopefully shed light on other IMS proteins that undergo post-translational modification to aid their import.

## 5. Alternative IMS import pathways

### 5.1. The stop-transfer pathway

**Figure 3.**
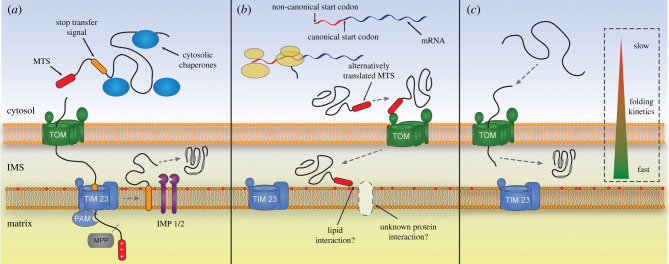
Alternative protein import pathways to the mitochondrial IMS. Nuclear-encoded, Mia40-independent proteins destined for the IMS are imported and retained in a number of different ways. (*a*) Proteins using the stop-transfer pathway (e.g. cytochrome *b*_2_) contain a bipartite signal composed of a positively charged mitochondrial targeting signal (MTS) at the proteins N-terminus followed by a hydrophobic segment. The MTS is targeted through the translocon of the outer membrane (TOM) and the translocon of the inner membrane (TIM23) into the matrix via the presequence translocase-associated motor (PAM). Further translocation to the matrix is blocked when the stop-transfer hydrophobic signal enters TIM23 and causes translocational arrest followed by lateral diffusion of this segment into the inner membrane. The MTS is cleaved by the mitochondrial processing peptidase (MPP) and a mature IMS protein is released via a second cleavage event mediated by IMS proteases such as IMP1/2. (*b*) Under stress conditions glutathione peroxidase 3 (Gpx3/Hyr1) is alternatively translated from a non-AUG start codon producing an extended protein containing a signal resembling an MTS. This extension enhances the mitochondrial localization of Gpx3 most likely by targeting Gpx3 via TOM, however whether this MTS interacts with TIM23 of some other inner membrane component is currently unknown. (*c*) Some slowly folding proteins in the cytosol can be transported into the IMS in an unfolded state, probably through the TOM, where increased folding kinetics leads to their retention in the IMS (e.g. Adk1 and Ccs1). Little is known however about the actual import pathway of this subset of IMS proteins.

The IMS is the destination for many nuclear-encoded proteins beyond simply substrates of the Mia import pathway. As such, further mechanisms are present for the import of proteins into the IMS. One of the first such mechanisms to be discovered and characterized was the stop-transfer pathway (for a recent review, see [55]). Nuclear-encoded proteins following this import route are synthesized with a bipartite presequence. The N-terminal part of the bipartite signal is a typical cleavable presequence similar to those found in proteins destined for the matrix. This positively charged, amphipathic helical structure is responsible for the initial targeting of the protein to the TOM complex through interactions with the cytosolic portions of receptors Tom20 and Tom22. Receptor binding facilitates outer membrane translocation through the Tom40 pore, a β-barrel protein related to the major outer membrane porin VDAC [39]. Subsequently, the presequence interacts with the large IMS region of the inner mitochondrial membrane receptor Tim50 and is fed through the Tim23 channel driven by the inner mitochondrial membrane potential (ΔΨ) [56]. At this stage, proteins destined for the matrix are translocated across the inner membrane by the presequence translocase-associated motor (PAM) complex driven by ATP hydrolysis, while proteins making use of the stop-transfer pathway remain in the IMS because of the second part (the ‘stop-transfer’ part) of the bipartite presequence that halts translocation at the level of the inner membrane. The ‘stop-transfer’ section of the bipartite presequence is a hydrophobic transmembrane segment immediately downstream of the presequence. This region is responsible for translocational arrest and retention of the protein in the IMS (figure 3*a*). The precise mechanism by which such a stop-transfer transmembrane segment can partition into the lipid bilayer has yet to be fully elucidated. However, the Tim23 channel seems to contain a lateral gate, much like other translocase channels such as the bacterial SecY and eukaryotic Sec61 translocases (for reviews, see [57,58]), that allows the direct partitioning of transmembrane regions from the channel into the hydrophobic core of the membrane [59]. In order to maintain fine control over the destination of presequence containing proteins, the TIM23 machinery adopts distinct conformations that aid either lateral diffusion into the inner membrane lipid bilayer or translocation into the matrix. Schendzielorz *et al.* [60] identified distinct conformations of TIM23 that underpin these two functions. These distinct conformations of the TIM23 complex involve interactions with Pam18 and Mgr2 and display differing lateral release phenotypes. Using a chimaeric version of Tim17–Pam18, they could show that Pam18 association the TIM23 complex (TIM23MOTOR) inhibited the lateral release of hydrophobic protein sequences, suggesting that Pam18 blocks the lateral gate of Tim23 [60]. A second conformation of the TIM23 complex containing Tim21 (TIM23SORT) is instead used for the partitioning of hydrophobic sequences into the inner membrane [61] aided by the gatekeeping protein Mgr2 [59,62]. Following lateral release, the matrix targeting presequence is cleaved by the matrix processing peptidase (MPP), while a second cleavage by the Imp1 or Imp2 proteases in the IMS releases the final mature protein domain in the IMS [56].

This prototypical stop-transfer pathway relates mainly to early work on cytochrome *b*2 and cytochrome *c*1, the most well-characterized stop-transfer substrates [63–65]. Further substrates with slightly altered characteristics have been identified in both human and yeast cells. Pro-apoptotic factors such as Aif [66], endonuclease G [67] and Smac/DIABLO [68] have all been identified in human cells as stop-transfer IMS proteins, although their import requirements have not been examined in detail [69]. The inner membrane protease PARL was however shown to process a Smac intermediate within the transmembrane stop-transfer sequence to yield the mature protein [70]. In yeast, the GTPase Mgm1 and the cytochrome *c* peroxidase Ccp1 contain a unique stop-transfer-like mechanism. Both proteins contain two transmembrane domains downstream of matrix-like targeting signals and require multiple protease cleavage events to mature correctly [71,72]. Mgm1 actually exists in two forms, a long and a short isoform, both essential for mitochondrial morphology and fusion. The stoichiometry of these two essential protein isoforms seems to be controlled by the levels of ATP within the matrix, with low levels leading to accumulation of the long isoform and aberrant mitochondrial morphology due to incomplete transport of the N-terminal presequence domain into the matrix [71]. The *S. cerevisiae* NADH dehydrogenase 1 (Nde1) was recently shown by Saladi *et al.* [73] to be targeted to the IMS via a transmembrane stop-transfer sequence, although no processing within the IMS seemed to occur with the protein remaining attached to the inner membrane. Nde1 is dually localized in both the IMS and the cytosolic face of the outer membrane. Interestingly, a reduction in the mitochondrial inner membrane potential increases the cytosolic localization of the protein and enhances proteasomal degradation of the soluble domain of Nde1 leading to apoptotic cell death. Interestingly, the transmembrane stop-transfer domain seemed to be embedded in the inner membrane even when the membrane potential was completely dissipated prior to import [73]. The *S. cerevisiae* type 2C protein phosphatase Ptc5 was identified in a recent proteomic study as a component of the IMS and contains a hydrophobic stop-transfer domain that is processed by Imp1 [2]. Stehlik *et al*. [74] recently showed that Ptc5 is dually localized to both the IMS and the peroxisome and that peroxisomal localization was dependent on initial mitochondrial targeting and IMS retention by the stop-transfer sequence, as well as processing by Imp1. The exact mechanism of how Ptc5 can exit the IMS or is stalled prior to complete entry remains to be elucidated but likely involves an interaction between the C-terminal peroxisome targeting motif of Ptc5 and the peroxisomal cytosolic receptor Pex5 [74].

### 5.2. A myriad of IMS proteins with atypical or unknown import pathways

#### 5.2.1. Cytochrome *c*/cytochrome *c* haem lyase

Many IMS proteins are not substrates of the Mia import pathway and do not contain a stop-transfer sequence. This means that they must be imported into the IMS via other, unconventional pathways. The most well characterized of these is the route taken by apo-cytochrome *c* and CCHL. Cytochrome *c* is localized on the IMS side of the inner mitochondrial membrane and acts as an electron carrier, transferring electrons from complex III to complex IV of the respiratory electron transport chain [75]. Apo-cytochrome *c* is translocated across the outer membrane via the Tom40 pore. Once in the IMS it accepts a haem cofactor from the IMS-localized CCHL resulting in the functional form of the holoenzyme cytochrome *c* [76]. The incorporation of the haem cofactor results in stably folded cytochrome *c* which, unlike the unfolded apo-protein, is prevented from retro-translocation through the Tom40 pore into the cytosol [77]. CCHL import is facilitated by the outer mitochondrial membrane receptor Tom20, which recognizes an internal hydrophobic targeting signals within CCHL [78,79]. The import of apo-cytochrome *c* on the other hand is independent from either the Tom20 or Tom70 receptors but dependent on Tom22, possibly due to the IMS-localized segment of Tom22 [80,81]. Apo-cytochrome *c* has also been shown to directly interact with lipids which perhaps make up for a requirement for the cytoplasmic Tom20 and Tom70 receptors [80]. The energetic driving force for outer membrane translocation of both apo-cytochrome *c* and CCHL remains to be resolved but their import does not involve ATP hydrolysis or Δ*Ψ* across the inner membrane.

#### 5.2.2. Adk1

In *S. cerevisiae*, adenylate kinase Adk1 is dually localized in both the cytosol and the mitochondrial IMS. This protein is a phosphotransferase which plays a crucial role in oxidative metabolism by converting ADP to ATP in the IMS [82]. A difference in the folding kinetics of the protein in the cytosol and the IMS has been implicated as a critical factor in the retention within the IMS [83]. However, the N-terminus may also act as a weak mitochondrial targeting signal that interacts with outer membrane receptors, such as Tom20 or Tom70 [84]. Internal targeting signals are likely to also play a role as some import of Adk1 into the IMS can still be observed even when the weak N-terminal targeting signal is removed [85].

#### 5.2.3. Gpx3

The yeast glutathione peroxidase Gpx3 (also known as Orp1 and Hyr1 [86]) is another protein which displays a non-conventional import pathway. *S. cerevisiae* encodes three glutathione peroxidases (Gpx1, Gpx2 and Gpx3) while humans encode eight (Gpx1–8). These proteins are essential for scavenging H_2_O_2_ and therefore reducing potentially harmful effects of ROS, especially in the generation of lipid peroxides which affect membrane stability and permeability. Using the term Gpx to describe these enzymes is not strictly true given that they do not depend on glutathione for their function, in reality they are thiol peroxidases which use internal thiols to detoxify H_2_O_2_ [87]. Yeast Gpx1 is loosely associated with the cytosolic leaflet of the outer mitochondrial membrane where it acts as a lipid peroxidase to detoxify outer membrane lipid peroxides [88]. Yeast Gpx2, on the other hand, is associated with the matrix leaflet of the inner mitochondrial membrane where it also functions as a lipid peroxide scavenger [89]. Yeast Gpx3 is the major cytosolic peroxide sensor which acts as a stress response transducer interacting with and activating the translation factor Yap1 [87]. A recent proteomic analysis identified Gpx3 as a component of the IMS suggesting a novel mitochondrial import mechanism [2]. Under hydrogen peroxide stress, the translation of Gpx3 is initiated at an upstream non-AUG start codon generating an N-terminally extended peptide exhibiting increased import into the mitochondrial IMS (figure 3*b*) [30]. The role of Gpx3 in the IMS is yet to be elucidated although mutants do display abnormal morphology and reduced membrane potential phenotypes. It seems logical to think that Gpx3 acts, at least partially, as a lipid peroxidase at the IMS leaflet of the inner membrane in a similar fashion to Gpx2 on the matrix side of the membrane. It is noteworthy that non-extended Gpx3 is also IMS-localized regardless of increased hydrogen peroxide stress, however its import pathway remains to be elucidated [30].

#### 5.2.4. Prx1

Yeast peroxiredoxin (Prx1) is a thioredoxin peroxidase first identified in the mitochondrial matrix and acts as a H_2_O_2_ scavenger [90]. Prx1 contains an N-terminal mitochondrial targeting sequence which is cleaved by MPP in the matrix. The mature protein is formed following a subsequent peptidase cleavage event in the matrix facilitated by Oct1 [91]. An IMS-localized version of Prx1 was recently identified which was released into the IMS by cleavage of the N-terminal signal sequence by the IMS protease Imp2. This localization seems to be dependent on a mildly hydrophobic stretch of amino acids at the end of the presequence, resulting in partitioning into the inner membrane following arrest in the Tim23 channel as opposed to full translocation into the matrix, a mechanism similar to the stop-transfer process [92].

#### 5.2.5. Sod1/Ccs1

The Cu/Zn superoxide dismutase (Sod1) catalyses the conversion of highly destructive superoxide radicals (O^−^) into hydrogen peroxide. The majority of cellular Sod1 resides in the cytosol, but a small fraction is also found in the IMS alongside the dedicated Sod1 chaperone Ccs1 which provides a copper ion and introduces a disulfide bond in Sod1, both of which are required for the Sod1 enzyme maturation [93]. While the levels of Sod1 and Ccs1 in the IMS are small in comparison to steady-state levels in the cytosol, the small volume of the IMS probably means the local concentration in this sub-compartment is actually very high as might be expected due to the large quantities of O^−^ released into the IMS by the respiratory electron transport chain. The import pathway of Sod1 remains unknown but likely follows a similar pathway to that proposed for Trx/Trr (see below). The IMS levels of Ccs1 are the limiting factor for Sod1 activity and the import pathway for Ccs1 has been relatively well characterized in both yeast and humans. In yeast, unfolded Ccs1 is a substrate for the Mia40 import pathway. Slow folding kinetics in the cytosol allow the unfolded form to be imported and retained in the IMS (figure 3*c*) [50,94]. In human cells, Ccs1 is not imported via the Mia40 (CHCHD4 in humans) pathway but instead requires a cytosolic version of itself for mitochondrial import. Once in the IMS, ROS levels determine the oxidative folding rate of Ccs1, and therefore its retention in this compartment [95].

#### 5.2.6. Trx1/Trr1

The yeast thioredoxin Trx1 is an important enzyme involved in the reduction of protein disulfide bonds for the maintenance of cellular redox homeostasis. Trx1 contains catalytic cysteine residues that transfer electrons to oxidized substrates breaking intramolecular disulfides. Reduction of oxidized Trx1 is then facilitated by thioredoxin reductase Trr1 which shuttles electrons from NADPH [86,96]. Both Trx1 and Trr1 have been identified in both the cytosol and the mitochondrial IMS although their mitochondrial import pathway remains unknown. Neither protein contains a mitochondrial presequence, although the presence of multiple cysteines, combined with their small size may implicate an oxidative folding mechanism (for example, via Mia40) as being responsible for their retention in the IMS [33].

#### 5.2.7. Ynk1

Yeast nucleoside diphosphate kinase Ynk1 exhibits dual localization between the cytosol and the mitochondrial IMS and exhibits a unique import mechanism. Ynk1 needs to be both unfolded and unphosphorylated in order to be imported through direct interactions with the Tom40 channel at the outer membrane, although a specific targeting signal is yet to be elucidated [97]. It is possible that the inclusion of a negatively charged phosphate within a targeting region alters the charge balance and thus interactions with the acidic patches on the surface of the Tom40 pore [39]. The function of Ynk1 in the IMS remains enigmatic although it has been hypothesized that it supplies GTP for mitochondrial biogenesis [98].

While many of the described import pathways have been relatively well characterized, the energy source(s) driving the import of many of these IMS proteins remains largely ambiguous. Several proteins containing a stop-transfer signal rely on the inner mitochondrial membrane potential for their maturation as might be expected due to the translocation of an N-terminal targeting sequence into the matrix, for example cytochrome *b*2 and Ptc5. However, other stop-transfer signal containing proteins like Nde1 do not require the inner membrane potential. Even less is known on the energetic mechanisms that facilitate transport into the IMS for unconventionally imported proteins that do not have a stop-transfer signal. It is possible that many dually localized proteins enter the IMS due to slow folding kinetics in the cytosol and are subsequently folded in the constricted environment of the IMS allowing their retention. Another intriguing possibility is that some proteins may be able to traverse the outer membrane in a partially folded state through interactions with currently unknown translocase complexes in the outer membrane, in a similar manner to the recently discovered AAA-ATPase Bcs1-Rieske protein translocation mechanism at the inner mitochondrial membrane [99] (figure 3).

## 6. IMS proteins in disease

Numerous diseases are caused by dysfunctional proteins within the IMS. Although a relatively constrained compartment with a small volume, the IMS contains a number of proteins critical for proper mitochondria and cell function. The variation in disease phenotypes from IMS protein dysfunction mirrors the functional diversity within the IMS proteome and consolidates the importance of this sub-compartment in both mitochondrial and cellular function. This section will highlight some of the most well-studied examples of IMS proteins that are implicated in disease.

### 6.1. Diseases associated with Mia40 substrates

Several Mia40 (CHCHD4 in humans) substrate proteins have been implicated in a variety of human diseases [100–103]. The human homologue of Erv1, called ALR, is linked to multiple diseases. Three accounts of a familial R194H mutation in ALR have shown that this results in a mitochondrial myopathy that causes respiratory chain deficiency, dystonia, deafness and lactic acidosis [104–106]. At a cellular level the reason for these effects is likely the failure of R194H ALR to import into the IMS which accumulates instead in the cytosol. Although there is a cytosolic form of ALR in addition to the mitochondrial form, this over-accumulation in the cytosol results in toxic effects, while the absence of ALR from the IMS causes a drastic dysfunction to the CHCHD4 pathway as reoxidation of CHCHD4 is impaired. Furthermore, ALR is thought to accelerate the progression of hepatocellular carcinomas although the molecular mechanism is yet to be elucidated [107].

Many of the small twin CX3C and CX9C containing proteins are attributed to diseases [101,108–110]. The IMS chaperone component Timm8A (DDP1) is responsible for the import of the Tim23 protein implicated in the assembly of the respiratory complex IV in neuronal cells [110,111]. Mutations in DDP1 result in the Mohr-Tranebjaerg syndrome which causes neurodegeneration, dystonia and deafness [112–116]. The cause of this condition is a mutation in one of the structural CX3C motif cysteines to a tryptophan (C66W) [117,118]. As this cysteine docks to Mia40 to form an intermolecular disulfide bond, lack of this cysteine residue results in abolished import of DDP1. The knock- on effect of this is dysfunction of the Tim23 import translocase which in turn determines the import of a plethora of inner membrane and matrix localized proteins. The neurodegenerative phenotype seen in patients with mutations in DDP1 could also be caused by assembly issues with complex IV as DPP1 knockout cells show depleted assembled complex IV levels [110]. In neuronal cells, DDP1 interacts with the complex IV assembly factors Cox17, Cox6B1, Coa7 and Coa4 and aids in the assembly of complex IV [110]. DDP1 (C66W) mutant cells have decreased complex IV, increased oxidative stress levels and elevated pro-apoptotic factors. This effect could be partially rescued with vitamin E supplementation which can detoxify lipid peroxides probably produced by dysfunctional complex IV. Mutations in a second CHCHD4 substrate and Complex IV assembly factor, COA6, are responsible for a neonatal hypertrophic cardiomyopathy that causes a severe complex IV deficiency [119,120]. The mutation of a tryptophan to arginine at position 66 (W66R) is likely to cause import and/or structural defects to the protein. Although there are a relatively small number of known CHCHD4 substrates that are involved in respiratory chain biogenesis it is intriguing that mutations in some of them cause several pathologies, in many cases linked to assembly defects of the cytochrome oxidase (COX) complex. The COX assembly factor COA7 is such an example. A patient with heterozygous mutations in COA7 resulted in no detectable COA7, a complex IV deficiency and neurological impairment [121]. Interestingly, a second study showed that the reason that no COA7 could be detected was due to a lack of protein import and subsequent proteasomal degradation in the cytosol [122]. Mohanraj *et al.* showed that in isolated patient fibroblasts the complex IV deficiency could be reversed upon treatment with the proteasomal inhibitor MG132, this suggests that the mutant form of COA7 is capable of assembling complex IV when imported but the kinetics of import are too slow resulting in proteasomal degradation [122].

Two proteins with typical CX9C cysteine motifs called coiled-coil-helix-coiled-coil-helix domain containing protein 2 (CHCHD2) and 10 are linked to several neurodegenerative diseases such as Parkinson's disease (PD), AS, ALS and frontotemporal lobe dementia (FTD) [108,109,123,124]. Although both proteins are Mia40 substrates several studies have shown that many of the mutations linked to neurodegeneration are not located near the CHCH domain that is responsible for their import into the IMS [109,125]. Instead CHCHD2 appears to aggregate inside mitochondria resulting in increased oxidative stress and apoptosis [126]. CHCHD2 even has prion-like properties whereby the mutant form of the protein can cause the wild-type to precipitate. The function of CHCHD2 is not well understood. CHCHD10 on the other hand is thought to play a role in maintaining mitochondrial cristae junctions and has links with the diseases ALS and FTD [124,127].

One critical function of mitochondria is regulating calcium homeostasis within the cell. Two regulatory subunits of the calcium uniporter MCU (mitochondrial calcium uniporter) in the mitochondrial inner membrane are involved in this regulation, MICU1 and MICU2. Although MICU1 possesses a mitochondrial targeting sequence, CHCHD4 is responsible for the formation of an intermolecular disulfide bond between MICU1 and MICU2 which is important for both proteins function and stability. Numerous mutations have been identified within MICU1 and MICU2 which cause myopathy, neurological symptoms and mitochondrial disorders [128,129]. Interestingly, both MICU1 and MICU2 are thought to bind the abundant inner membrane phospholipid cardiolipin [130]. The fact that MCU requires cardiolipin for stability suggests that MCU may play a role in the pathogenesis of Barth syndrome, a condition that causes a reduction in cardiolipin [131]. Future studies will help address the specific role of cardiolipin on MICU1 and MICU2 function and assembly.

### 6.2. Other IMS proteins in disease

The IMS-facing protein apoptosis inducing factor (AIF) is one such example of an IMS protein implicated in disease that does not require Mia40 for its import. AIF is imported via the stop-transfer pathway (see §5.1) although its function is not completely understood [103,132]. AIF interacts with CHCHD4 and is thought to regulate the import and tethering of CHCHD4 to the inner membrane [13]. Several studies have found mutations within AIF which have resulted in various diseases such as neonatal mitochondriopathy and late-onset axonal polyneuropathy [133–135]. Many of these mutations result in mitochondrial DNA loss and respiratory complex deficiencies, particularly complexes III and IV. Mutations that destabilize AIF could in turn affect the function of CHCHD4 in import of several assembly factors for complexes I and IV. This could provide a plausible mechanism explaining the respiratory complex deficiencies associated with AIF mutations.

The protein OPA1 is one of the most common causes of autosomal dominant optic atrophy. OPA1 is a dynamin-related GTPase that is responsible for the stabilization of the mitochondrial network due to its promotion of membrane fusion [136,137]. A plethora of different mutations have been identified in the gene which results in several different disease manifestations often comprising of two or more of the following clinical features: optic atrophy, deafness, ataxia, myopathy, neuropathy and progressive external ophthalmoplegia [136]. Many of these features are attributed to several mitochondrial diseases highlighting the importance of OPA1 in mitochondrial homeostasis. A full summary of IMS proteins in disease can be found in table 1.

**Table 1.** IMS proteins in disease.

protein name	function	Mia40 substrate (Y/N)	mutation	related disease	references
ALR	disulfide relay	Y	R194H	mitochondrial myopathy and respiratory chain deficiency	[104–106]
Timm8a	IMS chaperone	Y	C66W	Mohr- Tranebjaerg syndrome	[110,112,113,115,116,118]
Coa5	complex IV assembly	Y	A53P	cardioencephalomyopethy	[138]
Coa6	complex IV assembly	Y	W59C, W66R	neonatal hypertrophic cardiomyopathy	[119,120]
Coa7	complexes I and IV assembly	Y	Y137C	mitochondrial leukoencephalopathy and cytochrome *c* oxidase deficiency	[121,122]
Cox6B1	complex IV assembly	Y	R19H	severe infantile encephalomyopathy and mitochondrial complex IV deficiency	[139]
NDUFB10	complex I assembly	Y	C107S	lactic acidosis and cardiomyopathy	[140]
CHCHD2	cristae junction maintenance	Y	T61I	PD, AS, ALS and FTD	[101,108,109,125,126]
CHCHD10	cristae junction maintenance	Y	P34S, V57E, G58R, S59L, G66S, G66V, C122R, E127K	PD, AS, ALS and FTD	[109,124,127,141,142]
Micu	mitochondrial calcium homeostasis	Y	homozygous deletion	myopathy, neurological symptoms and mitochondrial disorders	[128,129,131]
AIF	respiratory chain biogenesis	N	T260A, L344F, G360R, R422W, R422Q, R430C, R451Q, A472V, P475L, V498M, I591M	neonatal mitochondriopathy and late-onset axonal polyneuropathy	[103,133,134]
Opa1	mitochondrial membrane inner membrane fusion	N	G401D, R445H, G488R, A495 V, S545R	optic atrophy, deafness, ataxia, myopathy, neuropathy and progressive external ophthalmoplegia	[136]

## 7. Discussion

**Figure 4.**
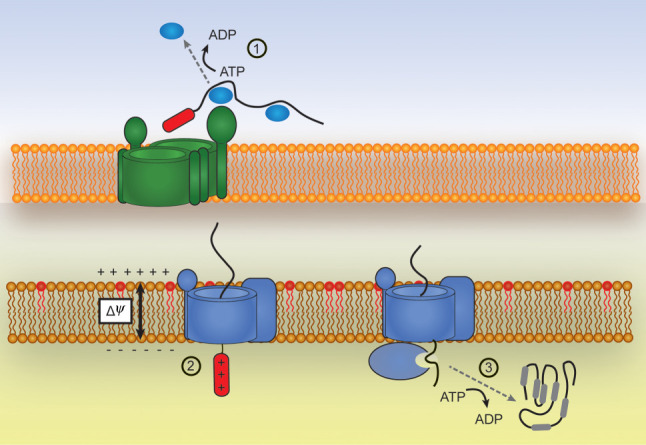
Energy inputs during mitochondrial protein import. The translocation of proteins across multiple lipid bilayer membranes, as is the case during mitochondrial import, requires energy inputs at various stages. (1) In order to pass across the outer mitochondrial membrane proteins often need to be maintained in an unfolded conformation by cytosolic chaperones such as Hsp70 and Hsp90. The first energy input often comes when these chaperones are released from the unfolded precursor via ATP hydrolysis at the outer surface of TOM. (2) A second energy input is required for initial translocation across the inner mitochondrial membrane. An intact membrane potential generated by proton pumping respiratory complexes is required for the translocation of positively charged MTS signals. (3) Further ATP hydrolysis steps are required for the further translocation of downstream protein segments into the matrix via the PAM motor. A number of IMS proteins show no dependence on membrane potential or ATP hydrolysis which leads to intriguing questions about the energy requirements for the outer membrane translocation of these proteins.

The mitochondrial proteome is encoded up to 99% by nuclear genes, and only 13 mitochondrial proteins are encoded by the mtDNA. The most recent analysis of the human mitochondrial proteome annotated a total of 1136 proteins in human mitochondria [4]. From these, only 53 proteins (5%) are localized in the IMS, whereas the other mitochondrial aqueous compartment, the matrix, houses almost 10 times as many proteins i.e. 525 polypeptides (46% of the total proteome). It is therefore very striking that although all matrix-targeted proteins are imported via one common pathway, targeting to the IMS is far more variable employing, as outlined in this review, an array of several different pathways. These IMS import pathways rely on (i) a variety of targeting peptides not sharing common features with each other and (ii) employ different sources of energy, which are neither the ATP hydrolysis in the matrix nor the inner membrane electrochemical potential (figure 4). For the majority of IMS-resident proteins retention in this compartment therefore depends on protein–protein, protein–ligand and, potentially, protein–lipid interactions.

The IMS is also very constricted in volume and highly segregated between the intra-cristae lumen which is separated by the rest of the IMS by the cristae junctions, and the bulk IMS which is delineated by the boundary IM and the OM. The small volume of the IMS combined with the internal compartmentalization of the IMS suggest that local protein concentrations may be very high. Consequently, the balance between productive folding and concentration-dependent aggregation is particularly critical for the biogenesis, function and homeostasis of proteins residing in this mitochondrial compartment. This concept is all the more important (and often not taken into consideration) given the increasing number of proteins that are found dually localized between the IMS and the cytosol. Two main classes of dedicated ATP-independent chaperones exist in the IMS to overcome potential problems of aggregation: (i) the small Tim chaperones that assist the passage across the IMS of membrane proteins en route to be inserted in the outer and inner membrane and (ii) the Mia40 protein that ensures the folding of many of the IMS-resident proteins. It would be interesting to investigate whether these chaperone systems have an additional role in the import and/or retention of dually localized proteins in the IMS. Furthermore, it will be exciting to explore the still elusive links between the internal mitochondrial structure (ensured by proteins like the MICOS complex that are not directly involved in protein import) and the IMS protein import pathways (figure 4).

## Data accessibility

This article has no additional data.

## References

[1] Herrmann JM, Riemer J. 2010 The intermembrane space of mitochondria. Antioxid. Redox Signal. **13**, 1341-1358. (10.1089/ars.2009.3063) 20367280

[2] Vögtle F-N et al. 2012 Intermembrane space proteome of yeast mitochondria. Mol. Cell. Proteomics **11**, 1840-1852. (10.1074/mcp.M112.021105) 22984289 PMC3518125

[3] Hung V, Zou P, Rhee H-W, Udeshi ND, Cracan V, Svinkina T, Carr SA, Mootha VK, Ting AY. 2014 Proteomic mapping of the human mitochondrial intermembrane space in live cells via ratiometric APEX tagging. Mol. Cell **55**, 332-341. (10.1016/j.molcel.2014.06.003) 25002142 PMC4743503

[4] Rath S et al. 2020 MitoCarta3.0: an updated mitochondrial proteome now with sub-organelle localization and pathway annotations. Nucleic Acids Res. **49**, D1541-D1547. (10.1093/nar/gkaa1011) PMC7778944 33174596

[5] Banci L, Bertini I, Ciofi-Baffoni S, Janicka A, Martinelli M, Kozlowski H, Palumaa P. 2008 A structural-dynamical characterization of human Cox17. J. Biol. Chem. **283**, 7912-7920. (10.1074/jbc.M708016200) 18093982

[6] Banci L, Bertini I, Ciofi-Baffoni S, Jaiswal D, Neri S, Peruzzini R, Winkelmann J. 2012 Structural characterization of CHCHD5 and CHCHD7: two atypical human twin CX9C proteins. J. Struct. Biol. **180**, 190-200. (10.1016/j.jsb.2012.07.007) 22842048

[7] Banci L, Bertini I, Cefaro C, Ciofi-Baffoni S, Gallo A, Martinelli M, Sideris DP, Katrakili N, Tokatlidis K. 2009 MIA40 is an oxidoreductase that catalyzes oxidative protein folding in mitochondria. Nat. Struct. Mol. Biol. **16**, 198-206. (10.1038/nsmb.1553) 19182799

[8] Chacinska A et al. 2004 Essential role of Mia40 in import and assembly of mitochondrial intermembrane space proteins. EMBO J. **23**, 3735-3746. (10.1038/sj.emboj.7600389) 15359280 PMC522791

[9] Hofmann S, Rothbauer U, Muhlenbein N, Baiker K, Hell K, Bauer MF. 2005 Functional and mutational characterization of human MIA40 acting during import into the mitochondrial intermembrane space. J. Mol. Biol. **353**, 517-528. (10.1016/j.jmb.2005.08.064) 16185709

[10] Mesecke N, Terziyska N, Kozany C, Baumann F, Neupert W, Hell K, Herrmann JM. 2005 A disulfide relay system in the intermembrane space of mitochondria that mediates protein import. Cell **121**, 1059-1069. (10.1016/j.cell.2005.04.011) 15989955

[11] Rissler M, Wiedemann N, Pfannschmidt S, Gabriel K, Guiard B, Pfanner N, Chacinska A. 2005 The essential mitochondrial protein Erv1 cooperates with Mia40 in biogenesis of intermembrane space proteins. J. Mol. Biol. **353**, 485-492. (10.1016/j.jmb.2005.08.051) 16181637

[12] Mordas A, Tokatlidis K. 2015 The MIA pathway: a key regulator of mitochondrial oxidative protein folding and biogenesis. Acc. Chem. Res. **48**, 2191-2199. (10.1021/acs.accounts.5b00150) 26214018 PMC4551283

[13] Hangen E et al. 2015 Interaction between AIF and CHCHD4 regulates respiratory chain biogenesis. Mol. Cell **58**, 1001-1014. (10.1016/j.molcel.2015.04.020) 26004228

[14] Meyer K, Buettner S, Ghezzi D, Zeviani M, Bano D, Nicotera P. 2015 Loss of apoptosis-inducing factor critically affects MIA40 function. Cell Death Dis. **6**, e1814. (10.1038/cddis.2015.170) 26158520 PMC4650723

[15] Endo T, Yamano K, Kawano S. 2010 Structural basis for the disulfide relay system in the mitochondrial intermembrane space. Antioxid. Redox Signal. **13**, 1359-1373. (10.1089/ars.2010.3099) 20136511

[16] Sideris DP, Petrakis N, Katrakili N, Mikropoulou D, Gallo A, Ciofi-Baffoni S, Banci L, Bertini I, Tokatlidis K. 2009 A novel intermembrane space-targeting signal docks cysteines onto Mia40 during mitochondrial oxidative folding. J. Cell Biol. **187**, 1007-1022. (10.1083/jcb.200905134) 20026652 PMC2806287

[17] Peleh V Cordat E, Herrmann JM. 2016 Mia40 is a trans-site receptor that drives protein import into the mitochondrial intermembrane space by hydrophobic substrate binding. Elife **5**, 937-944. (10.7554/eLife.16177) PMC4951193 27343349

[18] Koch JR, Schmid FX. 2014 Mia40 targets cysteines in a hydrophobic environment to direct oxidative protein folding in the mitochondria. Nat. Commun. **5**, 3041. (10.1038/ncomms4041) 24407114

[19] Koch JR, Schmid FX. 2014 Mia40 is optimized for function in mitochondrial oxidative protein folding and import. ACS Chem. Biol. **9**, 2049-2057. (10.1021/cb500408n) 24983157

[20] Chatzi A, Sideris DP, Katrakili N, Pozidis C, Tokatlidis K. 2013 Biogenesis of yeast Mia40: uncoupling folding from import and atypical recognition features. FEBS J. **280**, 4960-4969. (10.1111/febs.12482) 23937629

[21] Kallergi E et al. 2012 Targeting and maturation of Erv1/ALR in the mitochondrial intermembrane space. ACS Chem. Biol. **7**, 707-714. (10.1021/cb200485b) 22296668

[22] Bien M, Longen S, Wagener N, Chwalla I, Herrmann JM, Riemer J. 2010 Mitochondrial disulfide bond formation is driven by intersubunit electron transfer in Erv1 and proofread by glutathione. Mol. Cell **37**, 516-528. (10.1016/j.molcel.2010.01.017) 20188670

[23] Lionaki E, Aivaliotis M, Pozidis C, Tokatlidis K. 2010 The N-terminal shuttle domain of Erv1 determines the affinity for Mia40 and mediates electron transfer to the catalytic Erv1 core in yeast mitochondria. Antioxid. Redox Signal. **13**, 1327-1339. (10.1089/ars.2010.3200) 20367271

[24] Dabir DV, Leverich EP, Kim S-K, Tsai FD, Hirasawa M, Knaff DB, Koehler CM. 2007 A role for cytochrome c and cytochrome c peroxidase in electron shuttling from Erv1. EMBO J. **26**, 4801-4811. (10.1038/sj.emboj.7601909) 17972915 PMC2099471

[25] Bihlmaier K, Mesecke N, Terziyska N, Bien M, Hell K, Herrmann JM. 2007 The disulfide relay system of mitochondria is connected to the respiratory chain. J. Cell Biol. **179**, 389-395. (10.1083/jcb.200707123) 17967948 PMC2064786

[26] Tang X, Ang SK, Ceh-Pavia E, Heyes DJ, Lu H. 2020 Kinetic characterisation of Erv1, a key component for protein import and folding in yeast mitochondria. FEBS J. **287**, 1220-1231. (10.1111/febs.15077) 31569302 PMC7155059

[27] Ceh-Pavia E, Tang X, Liu Y, Heyes DJ, Zhao B, Xiao P, Lu H. 2020 Redox characterisation of Erv1, a key component for protein import and folding in yeast mitochondria. FEBS J. **287**, 2281-2291. (10.1111/febs.15136) 31713999 PMC7318334

[28] Neal SE, Dabir DV, Wijaya J, Boon C, Koehler CM. 2017 Osm1 facilitates the transfer of electrons from Erv1 to fumarate in the redox-regulated import pathway in the mitochondrial intermembrane space. Mol. Biol. Cell **28**, 2773-2785. (10.1091/mbc.E16-10-0712) 28814504 PMC5638582

[29] Schreiner B, Westerburg H, Forneb́ I, Imhof A, Neupert W, Mokranjac D. 2012 Role of the AAA protease Yme1 in folding of proteins in the intermembrane space of mitochondria. Mol. Biol. Cell **23**, 4335-4346. (10.1091/mbc.E12-05-0420) 22993211 PMC3496608

[30] Kritsiligkou P, Chatzi A, Charalampous G, Mironov A, Grant CM, Tokatlidis K. 2017 Unconventional targeting of a thiol peroxidase to the mitochondrial intermembrane space facilitates oxidative protein folding. Cell Rep. **18**, 2729-2741. (10.1016/j.celrep.2017.02.053) 28297675 PMC5368413

[31] Mesecke N, Bihlmaier K, Grumbt B, Longen S, Terziyska N, Hell K, Herrmann JM. 2008 The zinc-binding protein Hot13 promotes oxidation of the mitochondrial import receptor Mia40. EMBO Rep. **9**, 1107-1113. (10.1038/embor.2008.173) 18787558 PMC2581857

[32] Kojer K, Peleh V, Calabrese G, Herrmann JM, Riemer J. 2015 Kinetic control by limiting glutaredoxin amounts enables thiol oxidation in the reducing mitochondrial intermembrane space. Mol. Biol. Cell **26**, 195-204. (10.1091/mbc.E14-10-1422) 25392302 PMC4294668

[33] Habich M et al. 2019 Vectorial import via a metastable disulfide-linked complex allows for a quality control step and import by the mitochondrial disulfide relay. Cell Rep. **26**, 759-774; e5. (10.1016/j.celrep.2018.12.092) 30650365

[34] Bragoszewski P, Wasilewski M, Sakowska P, Gornicka A, Böttinger L, Qiu J, Wiedemann N, Chacinska A. 2015 Retro-translocation of mitochondrial intermembrane space proteins. Proc. Natl Acad. Sci. USA **112**, 7713-7718. (10.1073/pnas.1504615112) 26056291 PMC4485110

[35] Bragoszewski P, Gornicka A, Sztolsztener ME, Chacinska A. 2013 The ubiquitin-proteasome system regulates mitochondrial intermembrane space proteins. Mol. Cell. Biol. **33**, 2136-2148. (10.1128/MCB.01579-12) 23508107 PMC3648063

[36] Kurz M, Martin H, Rassow J, Pfanner N, Ryan MT. 1999 Biogenesis of Tim proteins of the mitochondrial carrier import pathway: differential targeting mechanisms and crossing over with the main import pathway. Mol. Biol. Cell **10**, 2461-2474. (10.1091/mbc.10.7.2461) 10397776 PMC25469

[37] Gornicka A, Bragoszewski P, Chroscicki P, Wenz LS, Schulz C, Rehling P, Chacinska A. 2014 A discrete pathway for the transfer of intermembrane space proteins across the outer membrane of mitochondria. Mol. Biol. Cell **25**, 3999-4009. (10.1091/mbc.E14-06-1155) 25318675 PMC4263444

[38] Araiso Y et al. 2019 Structure of the mitochondrial import gate reveals distinct preprotein paths. Nature **575**, 395-401. (10.1038/s41586-019-1680-7) 31600774

[39] Tucker K, Park E. 2019 Cryo-EM structure of the mitochondrial protein-import channel TOM complex at near-atomic resolution. Nat. Struct. Mol. Biol. **26**, 1158-1166. (10.1038/s41594-019-0339-2) 31740857 PMC8439582

[40] Edwards R, Tokatlidis K. 2019 The yeast voltage-dependent anion channel porin: more IMPORTant than just metabolite transport. Mol. Cell **73**, 861-862. (10.1016/J.MOLCEL.2019.02.028) 30849391

[41] Endo T, Sakaue H. 2019 Multifaceted roles of porin in mitochondrial protein and lipid transport. Biochem. Soc. Trans. **47**, 1269-1277. (10.1042/BST20190153) 31670371

[42] Doan KN, Ellenrieder L, Becker T. 2019 Mitochondrial porin links protein biogenesis to metabolism. Curr. Genet. **65**, 899-903. (10.1007/s00294-019-00965-z) 30944955

[43] Sakaue H et al. 2019 Porin associates with Tom22 to regulate the mitochondrial protein gate assembly. Mol. Cell **73**, 1044-1055. (10.1016/j.molcel.2019.01.003) 30738703

[44] Waegemann K, Popov-Čeleketić D, Neupert W, Azem A, Mokranjac D. 2015 Cooperation of TOM and TIM23 complexes during translocation of proteins into mitochondria. J. Mol. Biol. **427**, 1075-1084. (10.1016/j.jmb.2014.07.015) 25083920

[45] Nski O, Song J, Priesnitz C, Warscheid B, Pfanner N, Becker Correspondence T. 2018 Recruitment of cytosolic J-proteins by TOM receptors promotes mitochondrial protein biogenesis. CellReports **25**, 2036-2043. (10.1016/j.celrep.2018.10.083) PMC6280124 30463002

[46] Milenkovic D, Ramming T, Muller JM, Wenz LS, Gebert N, Schulze-Specking A, Stojanovski D, Rospert S, Chacinska A. 2009 Identification of the signal directing Tim9 and Tim10 into the intermembrane space of mitochondria. Mol. Biol. Cell **20**, 2530-2539. (10.1091/mbc.E08-11-1108) 19297525 PMC2682594

[47] Banci L et al. 2010 Molecular chaperone function of Mia40 triggers consecutive induced folding steps of the substrate in mitochondrial protein import. Proc. Natl Acad. Sci. USA **107**, 20 190-20 195. (10.1073/pnas.1010095107) PMC2996643 21059946

[48] Wrobel L, Trojanowska A, Sztolsztener ME, Chacinska A. 2013 Mitochondrial protein import: Mia40 facilitates Tim22 translocation into the inner membrane of mitochondria. Mol. Biol. Cell **24**, 543-554. (10.1091/mbc.E12-09-0649) 23283984 PMC3583659

[49] Longen S, Woellhaf MW, Petrungaro C, Riemer J, Herrmann JM. 2014 The disulfide relay of the intermembrane space oxidizes the ribosomal subunit Mrp10 on its transit into the mitochondrial matrix. Dev. Cell **28**, 30-42. (10.1016/j.devcel.2013.11.007) 24360785

[50] Groß DP, Burgard CA, Reddehase S, Leitch JM, Culotta VC, Hell K. 2011 Mitochondrial Ccs1 contains a structural disulfide bond crucial for the import of this unconventional substrate by the disulfide relay system. Mol. Biol. Cell **22**, 3758-3767. (10.1091/mbc.E11-04-0296) 21865601 PMC3192856

[51] Varabyova A, Topf U, Kwiatkowska P, Wrobel L, Kaus-Drobek M, Chacinska A. 2013 Mia40 and MINOS act in parallel with Ccs1 in the biogenesis of mitochondrial Sod1. FEBS J. **280**, 4943-4959. (10.1111/febs.12409) 23802566

[52] Reddehase S, Grumbt B, Neupert WHK. 2009 The disulfide relay system of mitochondria is required for the biogenesis of mitochondrial Ccs1 and Sod1. J. Mol. Biol. **385**, 331-338. (10.1016/j.jmb.2008.10.088) 19010334

[53] Weckbecker D, Longen S, Riemer J, Herrmann JM. 2012 Atp23 biogenesis reveals a chaperone-like folding activity of Mia40 in the IMS of mitochondria. EMBO J. **31**, 4348-4358. (10.1038/emboj.2012.263) 22990235 PMC3501227

[54] Ueda E, Tamura Y, Sakaue H, Kawano S, Kakuta C, Matsumoto S, Endo T. 2019 Myristoyl group-aided protein import into the mitochondrial intermembrane space. Sci. Rep. **9**, 1185. (10.1038/s41598-018-38016-1) 30718713 PMC6362269

[55] Pfanner N, Warscheid B, Wiedemann N. 2019 Mitochondrial proteins: from biogenesis to functional networks. Nat. Rev. Mol. Cell Biol. **20**, 267-284. (10.1038/s41580-018-0092-0) 30626975 PMC6684368

[56] Esaki M, Kanamori T, Nishikawa SI, Endo T. 1999 Two distinct mechanisms drive protein translocation across the mitochondrial outer membrane in the late step of the cytochrome b2 import pathway. Proc. Natl Acad. Sci. USA **96**, 11 770-11 775. (10.1073/pnas.96.21.11770) 10518525 PMC18361

[57] Tsirigotaki A, De Geyter J, Šoštarić N, Economou A, Karamanou S. 2017 Protein export through the bacterial Sec pathway. Nat. Rev. Microbiol. **15**, 21-36. (10.1038/nrmicro.2016.161) 27890920

[58] Lang S, Pfeffer S, Lee PH, Cavalié A, Helms V, Förster F, Zimmermann R. 2017 An update on Sec 61 channel functions, mechanisms, and related diseases. Front. Physiol. **8**, 887. (10.3389/fphys.2017.00887) 29163222 PMC5672155

[59] Ieva R et al. 2014 Mgr2 functions as lateral gatekeeper for preprotein sorting in the mitochondrial inner membrane. Mol. Cell **56**, 641-652. (10.1016/j.molcel.2014.10.010) 25454944

[60] Schendzielorz AB, Bragoszewski P, Naumenko N, Gomkale R, Schulz C, Guiard B, Chacinska A, Rehling P. 2018 Motor recruitment to the TIM23 channel's lateral gate restricts polypeptide release into the inner membrane. Nat. Commun. **9**, 4028. (10.1038/s41467-018-06492-8) 30279421 PMC6168564

[61] van der Laan M et al. 2007 Motor-free mitochondrial presequence translocase drives membrane integration of preproteins. Nat. Cell Biol. **9**, 1152-1159. (10.1038/ncb1635) 17828250

[62] Matta SK, Kumar A, D'Silva P. 2020 Mgr2 regulates mitochondrial preprotein import by associating with channel-forming Tim23 subunit. Mol. Biol. Cell **31**, 1112-1123. (10.1091/mbc.E19-12-0677) 32186971 PMC7353164

[63] Gasser SM, Ohashi A, Daum G, Bohni PC, Gibson J, Reid GA, Yonetani T, Schatz G. 1982 Imported mitochondrial proteins cytochrome-b2 and cytochrome-c1 are processed in 2 steps. Proc. Natl Acad. Sci. USA **79**, 267-271. (10.1073/pnas.79.2.267) 7043457 PMC345707

[64] Vanloon A, Brandli AW, Schatz G. 1986 The presequences of 2 imported mitochondrial proteins contain information for intracellular and intramitochondrial sorting. Cell **44**, 801-812. (10.1016/0092-8674(86)90846-9) 3004746

[65] Glick BS, Brandt A, Cunningham K, Muller S, Hallberg RL, Schatz G. 1992 Cytochromes-c1 and cytochromes-b2 are sorted to the intermembrane space of yeast mitochondria by a stop-transfer mechanism. Cell **69**, 809-822. (10.1016/0092-8674(92)90292-k) 1350514

[66] Susin SA et al. 1999 Molecular characterization of mitochondrial apoptosis-inducing factor. Nature **397**, 441-446. (10.1038/17135) 9989411

[67] Ohsato T, Ishihara N, Muta T, Umeda S, Ikeda S, Mihara K, Hamasaki N, Kang DC. 2002 Mammalian mitochondrial endonuclease G: digestion of R-loops and localization in intermembrane space. Eur. J. Biochem. **269**, 5765-5770. (10.1046/j.1432-1033.2002.03238.x) 12444964

[68] Burri L, Strahm Y, Hawkins CJ, Gentle IE, Puryer MA, Verhagen A, Callus B, Vaux D, Lithgow T. 2005 Mature DIABLO/Smac is produced by the IMP protease complex on the mitochondrial inner membrane. Mol. Biol. Cell **16**, 2926-2933. (10.1091/mbc.E04-12-1086) 15814844 PMC1142436

[69] Herrmann JM, Hell K. 2005 Chopped, trapped or tacked-protein translocation into the IMS of mitochondria. Trends Biochem. Sci. **30**, 205-211. (10.1016/j.tibs.2005.02.005) 15817397

[70] Saita S, Nolte H, Fiedler KU, Kashkar H, Venne AS, Zahedi RP, Krueger M, Langer T. 2017 PARL mediates Smac proteolytic maturation in mitochondria to promote apoptosis. Nat. Cell Biol. **19**, 318. (10.1038/ncb3488) 28288130

[71] Herlan M, Bornhovd C, Hell K, Neupert W, Reichert AS. 2004 Alternative topogenesis of Mgm1 and mitochondrial morphology depend on ATP and a functional import motor. J. Cell Biol. **165**, 167-173. (10.1083/jcb.200403022) 15096522 PMC2172034

[72] Michaelis G, Esser K, Tursun B, Stohn JP, Hanson S, Pratje E. 2005 Mitochondrial signal peptidases of yeast: the rhomboid peptidase Pcp1 and its substrate cytochrome c peroxidase. Gene **354**, 58-63. (10.1016/j.gene.2005.04.020) 15979251

[73] Saladi S et al. 2020 The NADH Dehydrogenase Nde1 executes cell death after integrating signals from metabolism and proteostasis on the mitochondrial surface. Mol. Cell **77**, 189. (10.1016/j.molcel.2019.09.027) 31668496

[74] Stehlik T, Kremp M, Kahnt J, Bolker M, Freitag J. 2020 Peroxisomal targeting of a protein phosphatase type 2C via mitochondrial transit. Nat. Commun. **11**, 2355. (10.1038/s41467-020-16146-3) 32398688 PMC7217942

[75] Wan J et al. 2019 Regulation of respiration and apoptosis by cytochrome c threonine 58 phosphorylation. Sci. Rep. **9**, 1-16. (10.1038/s41598-019-52101-z) 31676852 PMC6825195

[76] Dumont ME, Ernst JF, Hampsey DM, Sherman F. 1987 Identification and sequence of the gene encoding cytochrome-c heme lyase in the yeast *Saccharomyces-cerevisiae*. Embo J. **6**, 235-241. (10.1002/j.1460-2075.1987.tb04744.x) 3034577 PMC553382

[77] Mayer A, Neupert W, Lill R. 1995 Translocation of apocytochrome-c across the outer-membrane of mitochondria. J. Biol. Chem. **270**, 12 390-12 397. (10.1074/jbc.270.21.12390) 7759479

[78] Künkele K-P et al. 1998 The preprotein translocation channel of the outer membrane of mitochondria. Cell **93**, 1009-1019. (10.1016/S0092-8674(00)81206-4) 9635430

[79] Diekert K, Kispal G, Guiard B, Lill R. 1999 An internal targeting signal directing proteins into the mitochondrial intermembrane space. Proc. Natl Acad. Sci. USA **96**, 11 752-11 757. (10.1073/pnas.96.21.11752) 10518522 PMC18358

[80] Diekert K, de Kroon A, Ahting U, Niggemeyer B, Neupert W, de Kruijff B, Lill R. 2001 Apocytochrome c requires the TOM complex for translocation across the mitochondrial outer membrane. Embo J. **20**, 5626-5635. (10.1093/emboj/20.20.5626) 11598006 PMC125676

[81] Wiedemann N, Kozjak V, Prinz T, Ryan MT, Meisinger C, Pfanner N, Truscott KN. 2003 Biogenesis of yeast mitochondrial cytochrome c: a unique relationship to the TOM machinery. J. Mol. Biol. **327**, 465-474. (10.1016/s0022-2836(03)00118-9) 12628251

[82] Schricker R, Magdolen V, Kaniak A, Wolf K, Bandlow W. 1992 The adenylate kinase family in yeast: identification of URA6 as a multicopy suppressor of deficiency in major AMP kinase. Gene **122**, 111-118. (10.1016/0378-1119(92)90038-Q) 1333436

[83] Strobel G, Zollner A, Angermayr M, Bandlow W. 2002 Competition of spontaneous protein folding and mitochondrial import causes dual subcellular location of major adenylate kinase. Mol. Biol. Cell **13**, 1439-1448. (10.1091/mbc.01-08-0396) 12006643 PMC111117

[84] Agermayr M, Strobel G, Zollner A, Korber D, Bandlow W. 2001 Two parameters improve efficiency of mitochondrial uptake of adenylate kinase: decreased folding velocity and increased propensity of N-terminal alpha-helix formation. FEBS Lett. **508**, 427-432. (10.1016/S0014-5793(01)03122-2) 11728466

[85] Schricker R, Angermayr M, Strobel G, Klinke S, Korber D, Bandlow W. 2002 Redundant mitochondrial targeting signals in yeast adenylate kinase. J. Biol. Chem. **277**, 28 757-28 764. (10.1074/jbc.M201561200) 12045196

[86] Cardenas-Rodriguez M, Tokatlidis K. 2017 Cytosolic redox components regulate protein homeostasis via additional localisation in the mitochondrial intermembrane space. FEBS Lett. **591**, 2661-2670. (10.1002/1873-3468.12766) 28746987 PMC5601281

[87] Delaunay A, Pflieger D, Barrault M-B, Vinh J, Toledano MB. 2002 A thiol peroxidase is an H2O2 receptor and redox-transducer in gene activation. Cell **111**, 471-481. (10.1016/S0092-8674(02)01048-6) 12437921

[88] Inoue Y, Matsuda T, Sugiyama KI, Izawa S, Kimura A. 1999 Genetic analysis of glutathione peroxidase in oxidative stress response of *Saccharomyces cerevisiae*. J. Biol. Chem. **274**, 27 002-27 009. (10.1074/jbc.274.38.27002) 10480913

[89] Ukai Y, Kishimoto T, Ohdate T, Izawa S, Inoue Y. 2011 Glutathione peroxidase 2 in *Saccharomyces cerevisiae* is distributed in mitochondria and involved in sporulation. Biochem. Biophys. Res. Commun. **411**, 580-585. (10.1016/j.bbrc.2011.06.189) 21763276

[90] Pedrajas JR, Miranda-Vizuete A, Javanmardy N, Gustafsson JA, Spyrou G. 2000 Mitochondria of *Saccharomyces cerevisiae* contain one-conserved cysteine type peroxiredoxin with thioredoxin peroxidase activity. J. Biol. Chem. **275**, 16 296-16 301. (10.1074/jbc.275.21.16296) 10821871

[91] Voegtle FN et al. 2009 Global analysis of the mitochondrial n-proteome identifies a processing peptidase critical for protein stability. Cell **139**, 428-439. (10.1016/j.cell.2009.07.045) 19837041

[92] Gomes F, Palma FR, Barros MH, Tsuchida ET, Turano HG, Alegria TGP, Demasi M, Netto LES. 2017 Proteolytic cleavage by the inner membrane peptidase (IMP) complex or Oct1 peptidase controls the localization of the yeast peroxiredoxin Prx1 to distinct mitochondrial compartments. J. Biol. Chem. **292**, 17 011-17 024. (10.1074/jbc.M117.788588) PMC5641892 28821623

[93] Sturtz LA, Diekert K, Jensen LT, Lill R, Culotta VC. 2001 A fraction of yeast Cu,Zn-superoxide dismutase and its metallochaperone, CCS, localize to the intermembrane space of mitochondria: a physiological role for SOD1 in guarding against mitochondrial oxidative damage. J. Biol. Chem. **276**, 38 084-38 089. (10.1074/jbc.M105296200) 11500508

[94] Kloeppel C, Suzuki Y, Kojer K, Petrungaro C, Longen S, Fiedler S, Keller S, Riemer J. 2011 Mia40-dependent oxidation of cysteines in domain I of Ccs1 controls its distribution between mitochondria and the cytosol. Mol. Biol. Cell **22**, 3749-3757. (10.1091/mbc.E11-04-0293) 21865594 PMC3192855

[95] Suzuki Y, Ali M, Fischer M, Riemer J. 2013 Human copper chaperone for superoxide dismutase 1 mediates its own oxidation-dependent import into mitochondria. Nat. Commun. **4**, 2430. (10.1038/ncomms3430) 24026195

[96] Pedrajas JR, Kosmidou E, Miranda-Vizuete A, Gustafsson JA, Wright APH, Spyrou G. 1999 Identification and functional characterization of a novel mitochondrial thioredoxin system in *Saccharomyces cerevisiae*. J. Biol. Chem. **274**, 6366-6373. (10.1074/jbc.274.10.6366) 10037727

[97] Amutha B, Pain D. 2003 Nucleoside diphosphate kinase of *Saccharomyces cerevisiae*, Ynk1p: localization to the mitochondrial intermembrane space. Biochem. J. **370**, 805-815. (10.1042/bj20021415) 12472466 PMC1223228

[98] Petrungaro C, Riemer J. 2014 Mechanisms and physiological impact of the dual localization of mitochondrial intermembrane space proteins. Biochem. Soc. Trans. **42**, 952-958. (10.1042/bst20140104) 25109985

[99] Kater L, Wagener N, Berninghausen O, Becker T, Neupert W, Beckmann R. 2020 Structure of the Bcs1 AAA-ATPase suggests an airlock-like translocation mechanism for folded proteins. Nat. Struct. Mol. Biol. **27**, 142. (10.1038/s41594-019-0364-1) 31988523

[100] Modjtahedi N, Tokatlidis K, Dessen P, Kroemer G. 2016 Special issue: mitochondria and metabolism mitochondrial proteins containing coiled-coil-helix-coiled-coil-helix (CHCH) domains in health and disease. Trends Biochem. Sci. **41**, 245-260. (10.1016/j.tibs.2015.12.004) 26782138

[101] Zhou ZD, Saw WT, Tan EK. 2017 Mitochondrial CHCHD-containing proteins: physiologic functions and link with neurodegenerative diseases. Mol. Neurobiol. **54**, 5534-5546. (10.1007/s12035-016-0099-5) 27631878

[102] Jackson TD, Palmer CS, Stojanovski D. 2018 Mitochondrial diseases caused by dysfunctional mitochondrial protein import. Biochem. Soc. Trans. **46**, 1225-1238. (10.1042/BST20180239) 30287509

[103] Reinhardt C, Arena G, Nedara K, Edwards R, Brenner C, Tokatlidis K, Modjtahedi N. 2020 AIF meets the CHCHD4/Mia40-dependent mitochondrial import pathway. Biochim. Biophys. Acta Mol. Basis Dis. **1866**, 165746. (10.1016/j.bbadis.2020.165746) 32105825

[104] Di Fonzo A et al. 2009 The mitochondrial disulfide relay system protein GFER is mutated in autosomal-recessive myopathy with cataract and combined respiratory-chain deficiency. Am. J. Hum. Genet. **84**, 594-604. (10.1016/j.ajhg.2009.04.004) 19409522 PMC2681006

[105] Nambot S et al. 2017 Further delineation of a rare recessive encephalomyopathy linked to mutations in GFER thanks to data sharing of whole exome sequencing data. Clin. Genet. **92**, 188-198. (10.1111/cge.12985) 28155230

[106] Nalesnik MA, Gandhi CR, Starzl TE. 2017 Augmenter of liver regeneration: a fundamental life protein. Hepatology **66**, 266-270. (10.1002/hep.29047) 28085209 PMC5682950

[107] Gandhi CR et al. 2015 Liver-specific deletion of augmenter of liver regeneration accelerates development of steatohepatitis and hepatocellular carcinoma in mice. Gastroenterology **148**, 379-391.e4. (10.1053/j.gastro.2014.10.008) 25448926 PMC4802363

[108] Liu X, Jiao B, Zhang W, Xiao T, Hou L, Pan C, Tang B, Shen L. 2018 Identification of CHCHD2 mutations in patients with Alzheimer's disease, amyotrophic lateral sclerosis and frontotemporal dementia in China. Mol. Med. Rep. **18**, 461-466. (10.3892/mmr.2018.8962) 29749507

[109] Imai Y, Meng H, Shiba-Fukushima K, Hattori N. 2019 Twin CHCH proteins, CHCHD2, and CHCHD10: Key molecules of parkinson's disease, amyotrophic lateral sclerosis, and frontotemporal dementia. Int. J. Mol. Sci. **20**, 908. (10.3390/ijms20040908) 30791515 PMC6412816

[110] Kang Y et al. 2019 Function of hTim8a in complex IV assembly in neuronal cells provides insight into pathomechanism underlying mohr-tranebjærg syndrome. Elife **8**, e48828. (10.7554/eLife.48828) 31682224 PMC6861005

[111] Paschen SA, Rothbauer U, Kaldi K, Bauer MF, Neupert W, Brunner M. 2000 The role of the TIM8–13 complex in the import of Tim23 into mitochondria. EMBO J. **19**, 6392-6400. (10.1093/emboj/19.23.6392) 11101512 PMC305865

[112] Tranebjærg L et al. 2001 Neuronal cell death in the visual cortex is a prominent feature of the X-linked recessive mitochondrial deafness-dystonia syndrome caused by mutations in the TIMM8a gene. Ophthalmic Genet. **22**, 207-223. (10.1076/opge.22.4.207.2220) 11803487

[113] Tranebjærg L et al. 1995 A new X linked recessive deafness syndrome with blindness, dystonia, fractures, and mental deficiency is linked to Xq22. J. Med. Genet. **32**, 257-263. (10.1136/jmg.32.4.257) 7643352 PMC1050371

[114] Roesch K, Hynds PJ, Varga R, Tranebjaerg L, Koehler CM. 2004 The calcium-binding aspartate/glutamate carriers, citrin and aralar1, are new substrates for the DDP1/TIMM8a-TIMM13 complex. Hum. Mol. Genet. **13**, 2101-2111. (10.1093/hmg/ddh217) 15254020

[115] Jin H et al. 1996 A novel X–linked gene, DDP, shows mutations in families with deafness (DFN–1), dystonia, mental deficiency and blindness. Nat. Genet. **14**, 177-180. (10.1038/ng1096-177) 8841189

[116] Merchant SN, McKenna MJ, Nadol JB, Kristiansen AG, Tropitzsch A, Lindal S, Tranebjaeizrg L. 2001 Temporal bone histopathologic and genetic studies in Mohr-Tranebjaerg syndrome (DFN-1). Otol. Neurotol. **22**, 506-511. (10.1097/00129492-200107000-00017) 11449109

[117] Rothbauer U, Hofmann S, Mühlenbein N, Paschen SA, Gerbitz KD, Neupert W, Brunner M, Bauer MF. 2001 Role of the deafness dystonia peptide 1 (DDP1) in import of human Tim23 into the inner membrane of mitochondria. J. Biol. Chem. **276**, 37 327-37 334. (10.1074/jbc.M105313200) 11489896

[118] Hofmann S, Rothbauer U, Mühlenbein N, Neupert W, Gerbitz K-D, Brunner M, Bauer MF. 2002 The C66 W mutation in the deafness dystonia peptide 1 (DDP1) affects the formation of functional DDP1.TIM13 complexes in the mitochondrial intermembrane space. J. Biol. Chem. **277**, 23 287-23 293. (10.1074/jbc.M201154200) 11956200

[119] Calvo SE et al. 2012 Molecular diagnosis of infantile mitochondrial disease with targeted next-generation sequencing. Sci. Transl. Med. **4**, 118-128. (10.1126/scitranslmed.3003310) PMC3523805 22277967

[120] Baertling F et al. 2015 Mutations in *COA6* cause cytochrome *c* oxidase deficiency and neonatal hypertrophic cardiomyopathy. Hum. Mutat. **36**, 34-38. (10.1002/humu.22715) 25339201

[121] Lyons AM, Ardissone A, Reyes A, Robinson AJ, Moroni I, Ghezzi D, Fernandez-Vizarra E, Zeviani M. 2016 COA7 (C1orf163/RESA1) mutations associated with mitochondrial leukoencephalopathy and cytochrome c oxidase deficiency. J. Med. Genet. **53**, 846-849. (10.1136/jmedgenet-2016-104194) 27683825 PMC5264227

[122] Mohanraj K et al. 2019 Inhibition of proteasome rescues a pathogenic variant of respiratory chain assembly factor COA7. EMBO Mol. Med. **11**, e9561. (10.15252/emmm.201809561) 30885959 PMC6505684

[123] Klemann CJHM, Martens GJM, Sharma M, Martens MB, Isacson O, Gasser T, Visser JE, Poelmans G. 2017 Integrated molecular landscape of Parkinson's disease. npj Park. Dis. **3**, 14. (10.1038/s41531-017-0015-3) PMC5460267 28649614

[124] Bannwarth S et al. 2014 A mitochondrial origin for frontotemporal dementia and amyotrophic lateral sclerosis through CHCHD10 involvement. Brain **137**, 2329-2345. (10.1093/brain/awu138) 24934289 PMC4107737

[125] Yang N et al. 2019 Systematically analyzing rare variants of autosomal-dominant genes for sporadic Parkinson's disease in a Chinese cohort. Neurobiol. Aging **76**, 215.e1-215.e7. (10.1016/j.neurobiolaging.2018.11.012) 30598256

[126] Cornelissen T, Spinazzi M, Martin S, Imberechts D, Vangheluwe P, Bird M, De Strooper B, Vandenberghe W. 2020 CHCHD2 harboring Parkinson's disease-linked T61I mutation precipitates inside mitochondria and induces precipitation of wild-type CHCHD2. Hum. Mol. Genet. **29**, 1096-1106. (10.1093/hmg/ddaa028) 32068847

[127] Chaussenot A et al. 2014 Screening of CHCHD10 in a French cohort confirms the involvement of this gene in frontotemporal dementia with amyotrophic lateral sclerosis patients. Neurobiol. Aging **35**, 2884. (10.1016/j.neurobiolaging.2014.07.022) 25155093

[128] Logan CV et al. 2014 Loss-of-function mutations in MICU1 cause a brain and muscle disorder linked to primary alterations in mitochondrial calcium signaling. Nat. Genet. **46**, 188-193. (10.1038/ng.2851) 24336167

[129] Lewis-Smith D et al. 2016 Homozygous deletion in MICU1 presenting with fatigue and lethargy in childhood. Neurol. Genet. **2**, 1-6. (10.1212/NXG.0000000000000059) PMC4830195 27123478

[130] Kamer KJ, Grabarek Z, Mootha VK. 2017 High-affinity cooperative Ca^2+^ binding by MICU1-MICU2 serves as an on-off switch for the uniporter. EMBO Rep. **18**, 1397-1411. (10.15252/embr.201643748) 28615291 PMC5538426

[131] Ghosh S, Ball WB, Madaris TR, Srikantan S, Madesh M, Mootha VK, Gohil VM. 2020 An essential role for cardiolipin in the stability and function of the mitochondrial calcium uniporter. Proc. Natl Acad. Sci. USA **117**, 16 383-16 390. (10.1073/PNAS.2000640117) PMC7368250 32601238

[132] Otera H, Ohsakaya S, Nagaura Z-I, Ishihara N, Mihara K. 2005 Export of mitochondrial AIF in response to proapoptotic stimuli depends on processing at the intermembrane space. EMBO J. **24**, 1375-1386. (10.1038/sj.emboj.7600614) 15775970 PMC1142539

[133] Hu B, Wang M, Castoro R, Simmons M, Dortch R, Yawn R, Li J. 2017 A novel missense mutation in AIFM1 results in axonal polyneuropathy and misassembly of OXPHOS complexes. Eur. J. Neurol. **24**, 1499-1506. (10.1111/ene.13452) 28888069 PMC5693754

[134] Miyake N et al. 2017 X-linked hypomyelination with spondylometaphyseal dysplasia (H-SMD) associated with mutations in AIFM1. Neurogenetics **18**, 185-194. (10.1007/s10048-017-0520-x) 28842795 PMC5705759

[135] Zong L et al. 2015 Mutations in apoptosis-inducing factor cause X-linked recessive auditory neuropathy spectrum disorder. J. Med. Genet. **52**, 523-531. (10.1136/jmedgenet-2014-102961) 25986071 PMC4518735

[136] Yu-Wai-Man P et al. 2010 Multi-system neurological disease is common in patients with OPA1 mutations. Brain **133**, 771-786. (10.1093/brain/awq007) 20157015 PMC2842512

[137] Chan DC. 2007 Mitochondrial dynamics in disease. N. Engl. J. Med. **356**, 1707-1709. (10.1056/NEJMp078040) 17460225

[138] Huigsloot M et al. 2011 A mutation in C2orf64 causes impaired cytochrome c oxidase assembly and mitochondrial cardiomyopathy. Am. J. Hum. Genet. **88**, 488-493. (10.1016/j.ajhg.2011.03.002) 21457908 PMC3071910

[139] Massa V et al. 2008 Severe infantile encephalomyopathy caused by a mutation in COX6B1, a nucleus-encoded subunit of cytochrome C oxidase. Am. J. Hum. Genet. **82**, 1281-1289. (10.1016/j.ajhg.2008.05.002) 18499082 PMC2427282

[140] Friederich MW et al. 2017 Mutations in the accessory subunit NDUFB10 result in isolated complex I deficiency and illustrate the critical role of intermembrane space import for complex I holoenzyme assembly. Hum. Mol. Genet. **26**, 702-716. (10.1093/hmg/ddw431) 28040730 PMC6251674

[141] Lek M et al. 2016 Analysis of protein-coding genetic variation in 60,706 humans. Nature **536**, 285-291. (10.1038/nature19057) 27535533 PMC5018207

[142] Lehmer C et al. 2018 A novel CHCHD10 mutation implicates a Mia40-dependent mitochondrial import deficit in ALS. EMBO Mol. Med. **10**, e8558. (10.15252/emmm.201708558) 29789341 PMC5991575

